# Association between birth weight and risk of abdominal obesity in children and adolescents: a school-based epidemiology survey in China

**DOI:** 10.1186/s12889-020-09456-0

**Published:** 2020-11-10

**Authors:** Zhaogen Yang, Bin Dong, Yi Song, Xijie Wang, Yanhui Dong, Di Gao, Yanhui Li, Zhiyong Zou, Jun Ma, Luke Arnold

**Affiliations:** 1 Institute of Child and Adolescent Health & School of Public Health, Peking University, No. 38 Xueyuan Road, Haidian District, Beijing, 100191 People’s Republic of China; 2 Department of Commissioning, South Western Sydney Primary Health Network, Campbelltown, Australia

## Abstract

**Background:**

Abdominal obesity is becoming an increasingly serious public health challenge in children and adolescents, there remains controversial opinions on birth weight and risk of childhood abdominal obesity. This study aims to assess the association between birth weight and the risk of abdominal obesity in childhood, as well as to compare the associations among different sex and age groups.

**Methods:**

A total number of 30,486 (15,869 boys and 14,617 girls) participants aged 6–17 years old were included in this study. Participants were classified into five groups according to their birth weight. Waist-to-height ratio (WHtR) was used to define abdominal obesity. Fractional polynomial regression model was used to assess the association between birth weight and WHtR, and a multi-variable logistic regression model was applied to evaluate the risk of abdominal obesity in different birth weight groups.

**Results:**

A J-shaped association was observed between birth weight and WHtR. Compared with birth weight of 2500–2999 g, high birth weight was associated with increased risk of abdominal obesity [OR (95% CI) for 3000–3499 g: 1.12(1.00–1.24); 3500–3999 g: 1.19(1.07–1.34); ≥4000 g: 1.42(1.24–1.62)]. No significant correlation was observed in children with birth weight ≤ 2499 g. Similar patterns were observed across different age groups. Abdominal obesity risk for high birth weight was particularly pronounced in boys compared to girls.

**Conclusions:**

Birth weight ≥ 3000 g, especially for boys, was associated with an elevated risk of abdominal obesity in childhood and may benefit from intervention to mitigate this risk.

## Background

Childhood obesity is one of the most serious public health challenges worldwide [1]. In China, the prevalence of overweight and obesity among school-aged children has increased from 3.0% in 1985 to 19.4% in 2014 [2, 3]. It has been demonstrated that childhood overweight and obesity increase risk of many adverse health consequences throughout life-course [1, 4, 5].

Although obesity had been typically defined using body mass index (BMI), it has been demonstrated that BMI has limited capacity in measuring fat distribution [6]. Other anthropometric indices, such as waist-to-height ratio (WHtR) [7–9], are simple and useful indicators of abdominal fat. Compared with BMI, WHtR has been suggested as a superior predictor of obesity related adverse health consequences [10], including hypertension [11], metabolic syndrome [12], and other cardiovascular disease [13].

It has been suggested that early development (especially in utero) can influence a person’s physiology and metabolic health status permanently [14, 15]. Birth weight is considered as a proxy for fetal growth [16, 17]. Several previous studies have found significant associations between birth weight and abdominal obesity. Ansari [18] reported that high birth weight (> 4000 g) was associated with increased risk of abdominal obesity in children aged 6–18 years. Tian [19] and his colleague also found high birth weight (≥3500 g) to be a risk factor of abdominal obesity in people at 15–74 years old. Meanwhile, Vohr BR [20] reported that extreme preterm infants had increased risk of abdominal obesity when children entering into 6 to 7 years old. A study from southern England [21] had also found low birth weight to be associated with a tendency of central fat accumulation in 14 to 16 years old girls. However, some other studies [22, 23] did not observe significant correlations, and few had investigated this correlation with continuous variables, but the range of birth weight associated with the higher risk of abdominal obesity remained unclear.

It has also been proposed that the correlations may vary by sex and age. A cross-sectional study in 7 to 8 year-old Japanese children [24] has demonstrated a higher WHtR in girls with birth weight < 3000 g, but not in boys. Rodríguez Vargas N [22] found that high birth weight was not a predictive factor for abdominal obesity in 7–11 years old children, while another study found high birth weight to be a risk factor of abdominal obesity in 6 to 18 year-old children [18]. In summary, there was still a considerable lack of research regarding the effect of birth weight on the risk of childhood abdominal obesity. In this study, we aimed to explore the association between birth weight and WHtR in Chinese children and adolescents, and to assess the correlations between birth weight and risk of abdominal obesity by sex and age group.

## Methods

### Study design and data source

Data in this study comes from the baseline cross-sectional survey of a national multicenter cluster-controlled trial aimed to reduce the burden of obesity in children and adolescents (Registration number: NCT02343588). The survey was conducted in seven provinces or cities of China (Hunan, Ningxia, Tianjin, Chongqing, Liaoning, Shanghai, Guangzhou). Design and sampling procedure have been reported in detail elsewhere [25]. Briefly, a multi-stage cluster sampling method was used to select participants. At first, several regions were randomly selected from each province/city, and 12 to 16 schools were randomly chosen from each region. In each school, two classes were randomly selected in each grade and the whole class students were invited to participate in this survey. Those were recruited in this survey only after they and their parents signed informed consents voluntarily. All survey sites used the same protocol during the implementation process, and all processes of randomization were performed by a staff member who was not involved in the survey.

A total of 41,003 primary and middle school students aged 6–17 years old were enrolled in this study initially, of whom 30,486 remained in the analytical sample for the present work after excluding participants with missing records of urban/rural area (*n* = 234), weight (*n* = 1387), height (*n* = 1630), waist circumference (*n* = 1786), birthdate (*n* = 2780) or birth weight (*n* = 3902). The trial was approved by the Ethical Committee of Peking University (IRB0000105213034), and all participating children and their parents signed the informed consent forms for the physical examination and questionnaire survey.

### Anthropometric measurement

Anthropometric measurement was conducted by trained professional staff according to the standard protocol. Participants were asked to wear their underwear when waist circumference (WC, cm) and height (cm) were measured. WC was measured at the midpoint between the iliac crest and lowest rib by Myotape scale (Accufitness, Green Villge, Colordo, USA) with 0.1 cm precision, and height was measured using the portable stadiometer (model TZG, Jiangyin Hongya Science and Education Equipment Co., Ltd.) with 0.1 cm precision. Both WC and height were measured twice and the average of two records was used in data analysis. Waist-to-height ratio (WHtR) was calculated as WC divided by the height. The cut-off value of 0.5 was used for WHtR to classify abdominal obesity [26].

### Questionnaire survey

Students’ questionnaire and parental questionnaire were used to collect the information from children and their parents, respectively.

The students’ questionnaire was completed by themselves in children of grade 4 to 12 (with age range from 10 to 17 years old) and was completed under the assistance of their parents in children of grade 1 to 3 (with age range from 6 to 9 years old).

The students’ questionnaire was used to collect information of dietary behavior, including the consumption of fruits, vegetables, total meat, and sugar-sweetened beverages (SSBs) over the past 7 days. A serving of fruit/vegetable was defined as the size of an adult fist (approximately 120 g) [27], a serving of SSBs was defined as a canned beverage (approximately 250 ml), and a portion of meat equals to the size of an adult’s palm (approximately 100 g) [28]. Students reported the frequency (days) and amount (servings) of food and drink intake per day, the daily dietary intake was estimated as follow: average daily intake = (days × servings in those days) /7.

Information of child’s physical activity was recorded by the International Physical Activity Questionnaire-Short (IPAQ-S) [29], which has been widely used in children and adolescents. All recruited students were asked to report the frequency (days) and duration (hours and minutes) of moderate to vigorous-intensity physical activities (MVPA) over the past 7 days, and the average daily time of MVPA was calculated as follow: average daily time = (days × duration in those days)/7.

The parental questionnaire was filled out by parents at home. Paternal and maternal height and weight were reported in the parental questionnaire and then used to calculate body mass index (BMI), a cut-off point of BMI ≥ 24 kg/m^2^ was applied to define parental overweight/obesity. Parental educational level was classified into four categories (junior high school or below, senior high school, junior college, and college or above). Parents were required to report their children’s early life information, including birth weight, gestational age and number of fetus (singleton, twice or more) based on the birth certificate or health clinic record [30]. If they did not have it, parents were asked to recall the birth weight based on their own measurements. About 70.9% of parents report the information of their children’s early life based on the birth certificate or the health clinic card. Besides, information about feeding patterns (breastfeeding, not breastfeeding) and number of children (single child, two or more children) were additionally obtained. According to birth weight, participants were divided into five categories: < 2500 g, 2500-2999 g, 3000-3499 g, 3500-3999 g, and ≥ 4000 g [31].

### Statistical analysis

Descriptive results were characterized as means ± standard deviations for continuous variables and percentages for categorical variables. One-way ANOVA, independent sample Student’s t-test and Chi-square test were used to compare the distribution of descriptive characteristics between boys and girls. The nonlinear association between birth weight and WHtR was performed by fractional polynomial regression model. Logistic regression models were performed to assess the odds ratios (ORs) and 95% confidence intervals (CIs) for abdominal obesity when participants with birth weight of 2500–2999 g were used as the reference group. The crude model was adjusted for sex (only in total group), residence area, and age, while the adjusted model was further adjusted for gestational age, delivery mode, fetus number, feeding pattern, single-child status, paternal overweight/obesity, maternal overweight/obesity, paternal educational level, maternal educational level, daily fruit and vegetable consumption, daily meat consumption, daily SSBs consumption, and daily moderate to vigorous physical activity. Since about 25.6% of the original study sample had to be excluded for a variety of reasons, a sensitive analysis was conducted in the initial sample of 41,003 children. In addition, we have also done a sensitive analysis by extracting participants with original birth weight records (*n* = 21,615). All analyses were conducted using SPSS 20.0 (IBM, NY, USA) and Stata 14 software (College Station, TX, USA) and a two-sided *P* value of < 0.05 was considered statistically significant.

## Results

### Subject characteristics

A total of 30,486 students aged 6–17 years were included in the analysis. As showed in Table 1, 52.1% of the participants were boys, and 59.9% lived in urban areas. The mean birth weight was (3324.4 ± 502.9) g in total participants, which was higher in boys (3366.8 ± 513.9 g) than in girls (3278.5 ± 486.7 g) (*P*<0.01). The prevalence of abdominal obesity was 16.4% in total participants with a higher rate in boys (20.5%) than that in girls (12.0%) (*P*<0.05).

**Table 1:** The characteristics of Chinese children and adolescents aged 7–17 years

Variables	Total (*N* = 30,486)	Boys (*N* = 15,869)	Girls (*N* = 14,617)	*P* value
Residence area, N(%)				
Urban	18,255(59.9)	9557(60.2)	8698(59.5)	0.201
Rural	12,231(40.1)	6312(39.8)	5919(40.5)	
Age, years	10.9 ± 3.1	10.8 ± 3.1	11.0 ± 3.1	<0.001
Age group, N(%)				
6–9	12,252(40.2)	6515(41.1)	5737(39.2)	<0.001
10–13	11,818(38.8)	6159(38.8)	5659(38.7)	
14–17	6416(21.0)	3195(20.1)	3221(22.0)	
WC, cm	65.4 ± 10.9	66.6 ± 11.7	64.1 ± 9.8	<0.001
Height, cm	146.6 ± 16.4	147.7 ± 17.8	145.5 ± 14.6	<0.001
WHtR	0.45 ± 0.05	0.45 ± 0.06	0.44 ± 0.05	<0.001
Abdominal obesity, N (%)	5006(16.4)	3254(20.5)	1752(12.0)	<0.001
Birth weight, g	3324.4 ± 502.9	3366.8 ± 513.9	3278.5 ± 486.7	<0.001
Birth weight group, N (%)				
<2500	943(3.1)	460(2.9)	483(3.3)	<0.001
2500–2999	4057(13.3)	1865(11.8)	2192(15.0)	
3000–3499	13,504(44.3)	6649(41.9)	6855(46.9)	
3500–3999	8834(29.0)	4949(31.2)	3885(26.6)	
≥ 4000	3148(10.3)	1946(12.3)	1202(8.2)	
Gestational age, weeks	39.8 ± 1.2	39.7 ± 1.3	39.8 ± 1.2	<0.001
Fetus number, N (%)				
Singleton	29,421(97.4)	15,315(97.4)	14,106(97.4)	0.831
Twins or more	778(2.6)	408(2.6)	370(2.6)	
Breastfeeding, N (%)				
Breastfeeding	257 98(85.2)	13,371(84.9)	12,427(85.6)	0.075
Single child N (%)	20,371(66.8)	11,264(71.0)	9107(62.3)	<0.001
Paternal overweight/obesity, N (%)	15,052(49.4)	7953(50.1)	7099(48.6)	<0.001
Maternal overweight/obesity, N (%)	7076(23.2)	3632(22.9)	3444(23.6)	0.164
Paternal educational level, N (%)				
Junior high school or blew	12,948(42.7)	6780(43.0)	6168(42.4)	0.767
Senior high school	8468(27.9)	4395(27.9)	4073(28.0)	
Junior college	4465(14.7)	2310(14.7)	2155(14.8)	
College or above	4424(14.6)	2282(14.5)	2142(14.7)	
Maternal educational level, N (%)				
Junior high school or blew	14,214(47.0)	7454(47.4)	6760(46.5)	0.427
Senior high school	7940(26.2)	4076(25.9)	3864(26.6)	
Junior college	4462(14.7)	2305(14.6)	2157(14.9)	
College or above	3648(12.1)	1904(12.1)	1744(12.0)	
Daily fruit and vegetable consumption, servings	3.2 ± 2.1	3.2 ± 2.1	3.2 ± 2.0	0.638
Daily SSBs consumption, servings	0.4 ± 0.7	0.5 ± 0.8	0.3 ± 0.6	<0.001
Daily meat consumption, servings	1.2 ± 1.2	1.3 ± 1.3	1.0 ± 1.0	<0.001
Daily moderate to vigorous physical activity duration, hours	0.7 ± 1.0	0.8 ± 1.1	0.7 ± 1.0	<0.001

Notes: *WC* Waist circumference; *WHtR* Waist-to-height ratio; *SSBs* Sugar-sweetened beverages

### The nonlinear relationship between birth weight and WHtR

**Fig. 1:**
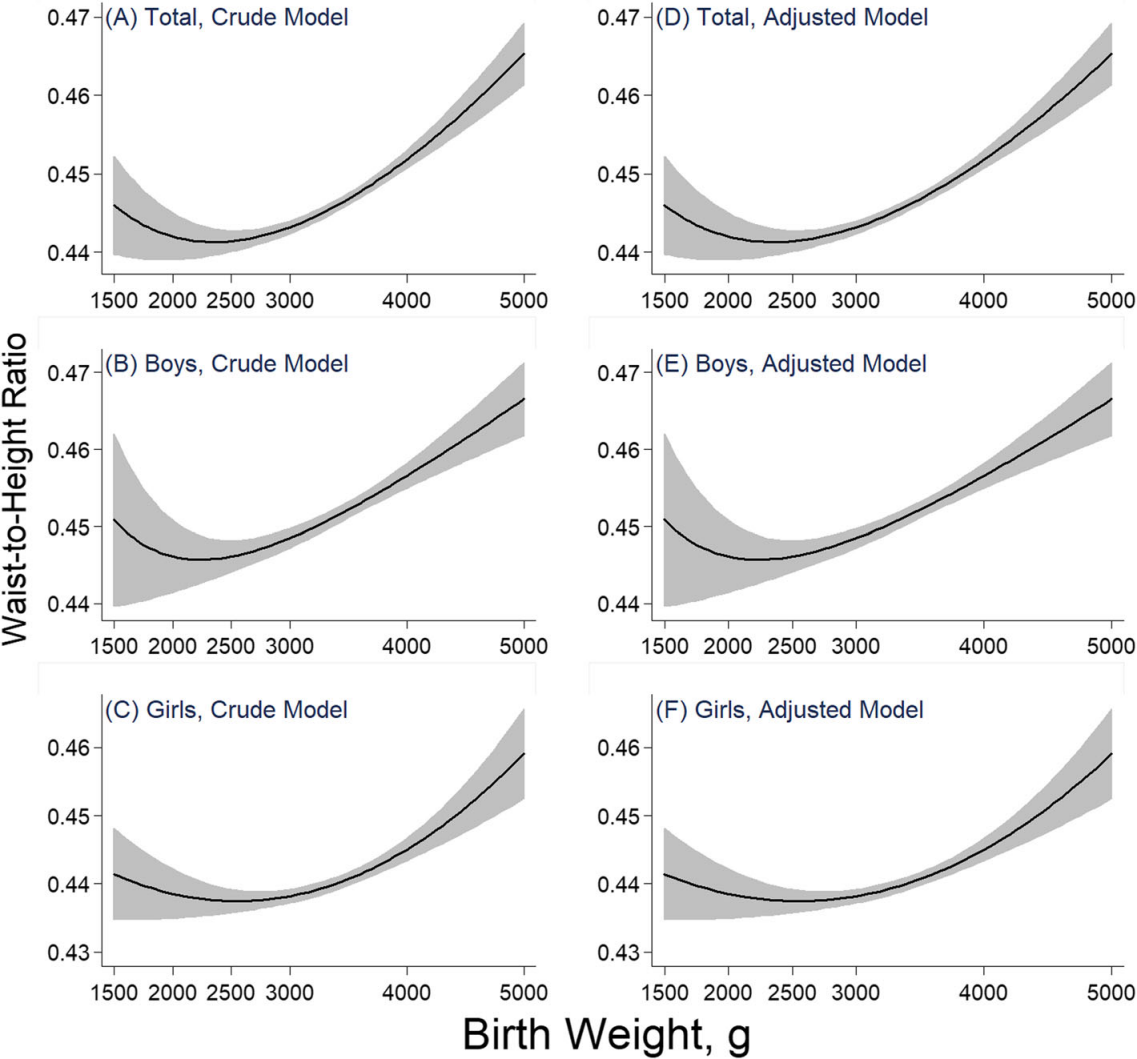
The relationship between birth weight and WHtR in children and adolescents. Notes: the crude model was adjusted for sex (only in total group), residence area, and age; the adjusted model was futher adjusted for gestational age, delivery mode, fetus number, feeding pattern, single-child or not, paternal overweight/obesity, maternal overweight/obesity, paternal educational level, maternal educational level, daily fruit and vegetable consumption, daily meat consumption, daily SSBs consumption, daily moderate to vigorous physical activity

The nonlinear relationship of birth weight and WHtR is shown in Fig. 1. A J-shaped association was observed in the crude model. This association was slightly changed after further adjusting for confounding factors, with the range of birth weight associated with the lowest WHtR around 2500 g. In the stratified analysis, similar patterns were detected both in boys and girls. However, the birth weight associated with the lowest WHtR in boys was lower than that in girls.

### Association between birth weight and risk of abdominal obesity in boys and girls

**Fig. 2:**
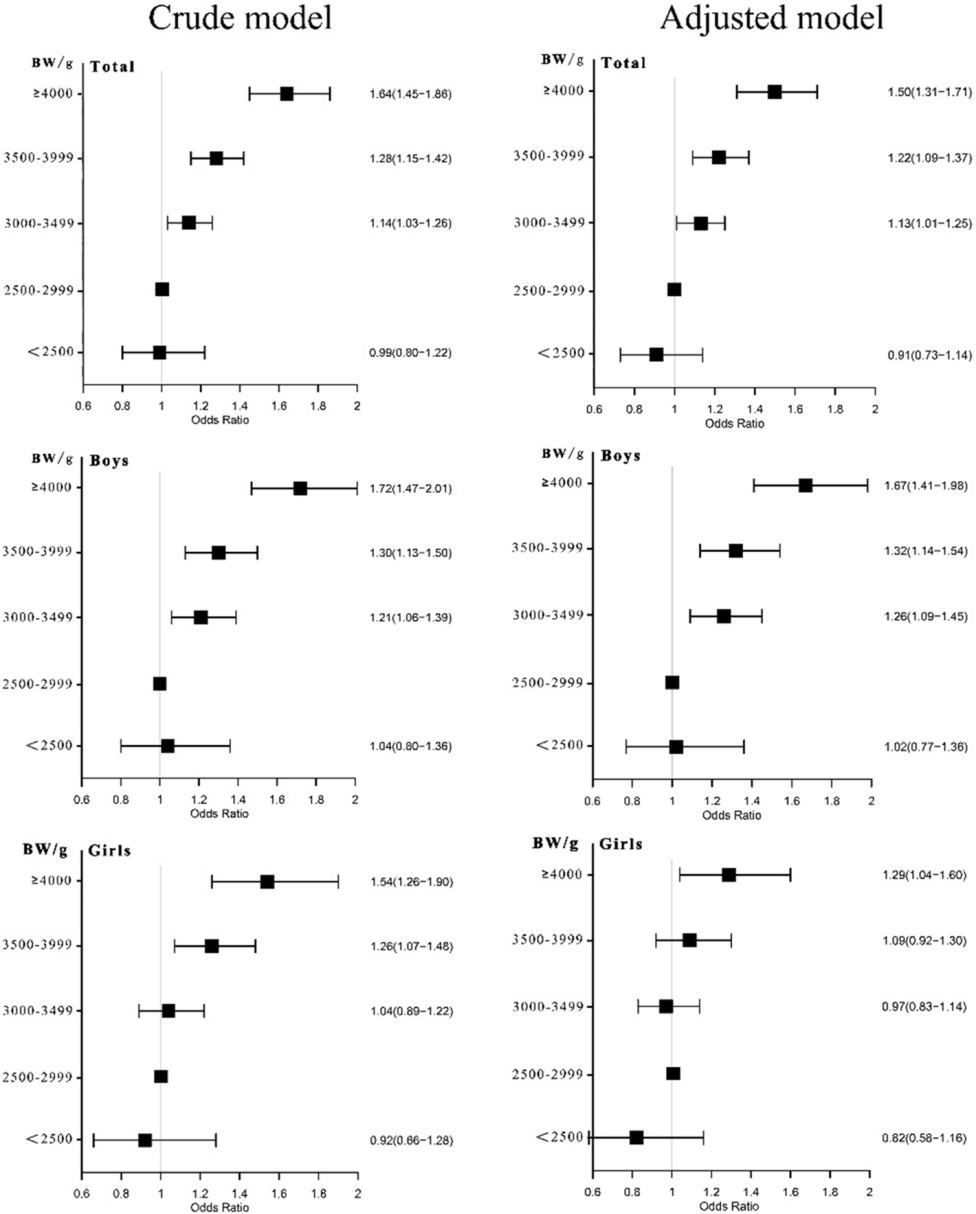
Risk of childhood abdominal obesity in boys and girls with different birth weights. Notes: crude model was adjusted for sex (only in total group), residence area, and age; the adjusted model was further adjusted for gestational age, fetus number, feeding pattern, single-child or not, paternal overweight/obesity, maternal overweight/obesity, paternal educational level, maternal educational level, daily fruit and vegetable consumption, daily meat consumption, daily SSBs consumption, daily moderate to vigorous physical activity on the basic of crude model

Figure 2 shows the association between birth weight and risk of abdominal obesity in boys and girls. The crude model shows that compared with participants whose birth weight ranged from 2500 to 2999 g, those who had higher birth weight (3000–3499 g, 3500–3999 g, ≥ 4000 g) are associated with an elevated risk of abdominal obesity. Though lower ORs were detected in participants with birth weight < 2500 g, especially in girls, the difference was not statistically significant (*P* = 0.692). When further adjusted for potential confounding factors, only minor changes were observed for these estimations. The birth weight (≥ 3000 g) was positively associated with higher risk of abdominal obesity (for 3000–3499 g: OR[95% CI] = 1.13[1.01,1.25]; for 3500–3999 g: OR[95% CI] = 1.22[1.09,1.37]; for ≥4000 g: OR[95% CI] **=** 1.50[1.31,1.71]). In addition, the ORs of abdominal obesity were higher in boys than those in girls in the same birth weight group. Participants had an evelated risk for those with a birth weight ≥ 3000 g in boys and ≥ 4000 g in girls. Based on the original sample (*n* = 41,003) and original birth record sample (*n* = 21,615), we have done two sensitive analyses and have come to similar results with the present study (Table S1 and Table S3).

### Association between birthweight and risk of abdominal obesity in different age groups

To investigate whether the association varied among age groups, participants were divided into three groups according to their ages. Table 2 shows that high birth weight was related to abdominal obesity regardless of the age groups. Participants with birth weight ≥ 4000 g had higher risk of abdominal obesity in all three age groups, with the ORs ranging from 1.27 (95% CI: 1.03–1.58) to 1.59 (95% CI: 1.29–1.97). Participants with birth weight between 3000 and 3999 g also showed an elevated risk, though the statistically significant result was only found in children aged 6–9 years. In addition, across the age groups, no significant association between low birth weight (< 2500 g) and abdominal obesity was found. Based on the original sample (*n* = 41,003) and original birth record sample (*n* = 21,615), we have done two sensitive analyses and have come to similar results with the present study. (Table S2 and Table S4).

**Table 2:** Associations between birth weight and childhood abdominal obesity in different age groups

Birth weight groups	6–9 years old			10–13 years old			14–17 years old		
	N	OR(95% CI)	*P* value	N	OR(95% CI)	*P* value	N	OR(95% CI)	*P* value
≥4000	1153	1.59(1.29–1.97)	<0.001	1175	1.27(1.03–1.58)	0.006	820	1.52(1.12–2.07)	0.006
3500–3999	3411	1.23(1.03–1.46)	0.009	3540	1.14(0.96–1.36)	0.082	1883	1.29(0.98–1.69)	0.055
3000–3499	5635	1.08(0.91–1.27)	0.297	5167	1.14(0.96–1.34)	0.099	2702	1.18(0.91–1.53)	0.202
2500–2999	1647	Reference		1600	Reference		810	Reference	
< 2500	406	0.81(0.58–1.15)	0.249	336	0.83(0.57–1.20)	0.340	201	1.40(0.87–2.26)	0.174

Notes: The model was adjusted for sex, residence area, gestational age, fetus number, feeding pattern, single-child or not, paternal overweight/obesity, maternal overweight/obesity, paternal educational level, maternal educational level, daily fruit and vegetable consumption, daily meat consumption, daily SSBs consumption and daily moderate to vigorous physical activity

## Discussion

In this study of 30,486 Chinese children and adolescents, the prevalence of abdominal obesity is 16.4%, the rate was lower than data from Spanish (21.3%) [32], but higher than results of Australian children in 2015 (14.6%) [33]. The reasons for the difference are complicated and variations of ethnicity, dietary behavior, lifestyle and economic development may contribute to it [32, 33]. Our study indicated that boys were more likely to have abdominal obesity than girls, which is in line with several previous studies [32, 33].

A J-shaped relationship was observed between birth weight and WHtR in this study. On the one hand, a positive association between birth weight and WHtR was found in children with birth weight > 3000 g, especially in boys. These findings were consistent with studies conducted by Schellong [34], Loaiza [35] and Kang [36]. One possible reason for this may be the sexual difference in growth of body composition, bone, and muscle growth during prenatal period [37, 38].

Additionally, the present study found that low birth weight (< 2500 g) was not significantly associated with risk of abdominal obesity regardless of sex and age. Studies from Frye [39], Yuan [31] and Singhal [40] had also demonstrated similar conclusion that low birth weight was not a risk factor of obesity, they point that children who born in unfavorable nutritional conditions may have permanent influence in later life. Yuan [31] found children with extremely low birth weight (less than 1500 g) had slightly higher risk of abdominal obesity, even though low birth weight (2000-2499 g, 1500-1999 g) was not significant related with abdominal obesity. A cohort study conducted by Vohr BR [20] et al. had also come to similar conclusions, in that extreme preterm infant had increased rate of abdominal obesity. One explanation is that infants who are born with a low birth weight tend to experience a later catch-up growth, which leads to higher risk of abdominal obesity in later years [41].

The association between birth weight and abdominal obesity may be explained by intrauterine environmental condition, genetic and lifestyle factors. Birth weight is regarded as a reflection of the intrauterine environmental situation [42]. On the one hand, over nutrition and malnutrition during pregnancy can have effects on birth weight, and this environmental exposure can induce persistent alterations in the epigenome, which leads to an increased risk of obesity in later life. On the other hand, it has been found that the number of muscle cells in the body is influenced by birth weight, that is, infants with higher birth weight had greater number of cells in both adipose and non-adipose tissues [43], which could persist later in life. Furthermore, genetic factors may also influence the fat accumulation in childhood. Sharp’s study [44] found that low birth weight affects the neonatal epigenome via DNA methylation. Further, Lin [45] suggested that developmental pathways to adiposity begin before birth and are influenced by genetic and epigenetic factors. Previous studies have also demonstrated that birth weight was associated with later exercise capacity or physical activity performance. Andersen and colleagues [46] found that adolescents with high birth weight had lower physical activity level.

Extending the findings of previous studies, we have further conducted an analysis by different age group. While high birth weight (> 4000 g) was significantly associated with abdominal obesity in all age groups, elevated risk of abdominal obesity was also observed in birth weight group of 3500–3999 g, but only in those of younger ages (6–9 years). A possible explanation for this is that the effects were influenced by other environmental factors which were not considered in this study, for example, it has been reported that younger children spend more time in doors than older children, and they are less sensitive to unhealthy food outside of the home than older children [47], and more environmental factors could affect abdominal obesity in adolescents within older age group [48].

There are several limitations in this study. First, the birth weight information was obtained from parental questionnaire, rather than directly from the hospital birth records database, and nearly 30% children’s birth weight were obtained by parental recall. Second, this study was based on a cross-sectional study, and no causal relationship can be suggested. In addition, we did not have the information of parental nutritional status during pregnancy. It has been suggested that gestational weight gain could lead to fetal macrosomia and obesity after birth. Future study is needed to determine the relationship between birth weight and other health consequences in later life.

## Conclusion

In summary, this study examined the association between birth weight and risk of abdominal obesity in Chinese children and adolescents aged 6–17 years old. A J-shaped relationship was found between birth weight and WHtR, and children with high birth weight (> 3000 g) had higher risk of abdominal obesity than those with low birth weight, especially in boys. This study would contribute to a better understanding of the relationship between intrauterine growth and abdominal obesity in later life. Our findings could aid the clinical practitioners to identify youth at risk of abdominal obesity, and have a potential to improve intervention strategies aimed at reducing the burden of childhood obesity. Further study is needed to explore the mechanisms underlying this association.

## Supplementary information

**Additional file 1: Table S1.** Risk of childhood abdominal obesity in boys and girls with different birth weights in Total participants (*N* = 41,003). **Table S2.** Associations between birth weight and childhood abdominal obesity in different age groups. **Table S3.** Risk of childhood abdominal obesity in boys and girls with different birth weights in participants with original birth weight records(*N*=21,615). **Table S4.** Associations between birth weight and childhood abdominal obesity in participants with original birth weight records, stratified by age groups

## Data Availability

The datasets generated during and/or analyzed during the current study are not publicly available due to confidentiality of data and subsequent research, but are available from the corresponding author on reasonable request.
