# Supplementary material for: Association between birth weight and risk of abdominal obesity in children and adolescents: a school-based epidemiology survey in China
Source: BMC Public Health. 2020 Nov 10;20:1686. doi: 10.1186/s12889-020-09456-0 (PMC7656713; doi:10.1186/s12889-020-09456-0)
Supplement: Supplementary file 1 — Additional file 1: Table S1. Risk of childhood abdominal obesity in boys and girls with different birth weights in Total participants (N = 41,003). Table S2. Associations between birth weight and childhood abdominal obesity in different age groups. Table S3. Risk of childhood abdominal obesity in boys and girls with different birth weights in participants with original birth weight records(N=21,615). Table S4. Associations between birth weight and childhood abdominal obesity in participants with original birth weight records, stratified by age groups [file 12889_2020_9456_MOESM1_ESM.docx]

| Table S1 Risk of childhood abdominal obesity in boys and girls with different birth weights in Total participants (N=41,003) | | | | | |
| --- | --- | --- | --- | --- | --- |
| Groups | Crude model | |  | Adjusted model | |
|  | OR (95%CI) | *P* value |  | OR (95%CI) | *P* value |
| Total |  |  |  |  |  |
| ≥4000 | 1.51(1.35,1.68) | < 0.001 |  | 1.62(1.43,1.84) | < 0.001 |
| 3500-3999 | 1.26(1.15,1.38) | < 0.001 |  | 1.30(1.17,1.44) | < 0.001 |
| 3000-3499 | 1.13(1.03,1.23) | 0.008 |  | 1.15(1.04,1.27) | 0.006 |
| 2500-2999 | Reference |  |  | Reference |  |
| <2500 | 1.03(0.87,1.22) | 0.743 |  | 1.01(0.83,1.23) | 0.945 |
| Boys |  |  |  |  |  |
| ≥4000 | 1.64(1.43,1.89) | < 0.001 |  | 1.62(1.37,1.91) | < 0.001 |
| 3500-3999 | 1.30(1.14,1.47) | < 0.001 |  | 1.32(1.14,1.52) | < 0.001 |
| 3000-3499 | 1.23(1.09,1.39) | 0.001 |  | 1.29(1.12,1.49) | < 0.001 |
| 2500-2999 | Reference |  |  | Reference |  |
| <2500 | 1.05(0.84,1.32) | 0.656 |  | 1.02(0.78,1.33) | 0.868 |
| Girls |  |  |  |  |  |
| ≥4000 | 1.36(1.14,1.61) | 0.001 |  | 1.31(1.07,1.59) | 0.008 |
| 3500-3999 | 1.23(1.07,1.40) | 0.003 |  | 1.11(0.95,1.29) | 0.200 |
| 3000-3499 | 1.01(0.89,1.14) | 0.928 |  | 0.94(0.82,1.09) | 0.438 |
| 2500-2999 | Reference |  |  | Reference |  |
| <2500 | 1.01(0.78,1.30) | 0.931 |  | 0.94(0.70,1.27) | 0.687 |
| Notes: crude model was adjusted for sex(only in total group), residence area, and age; the adjusted model was further adjusted for gestational age, fetus number, feeding pattern, single-child or not, paternal overweight/obesity, maternal overweight/obesity, paternal educational level, maternal educational level, daily fruit and vegetable consumption, daily meat consumption, daily SSBs consumption, daily moderate to vigorous physical activity on the basic of crude model. | | | | | |

| Table S2 Associations between birth weight and childhood abdominal obesity in different age groups | | | | | | | | |
| --- | --- | --- | --- | --- | --- | --- | --- | --- |
| Birth weight | 6-9 years old | | 10-13 years old | | |  | 14-17 years old | |
| groups | OR (95% CI) | *P* value |  | OR (95% CI) | *P* value |  | OR (95% CI) | *P* value |
| ≥4000 | 1.93(1.60,2.31) | < 0.001 |  | 1.51(1.23,1.86) | < 0.001 |  | 1.50(1.10,2.03) | 0.010 |
| 3500-3999 | 1.33(1.14,1.55) | < 0.001 |  | 1.23(0.98,1.47) | 0.096 |  | 1.33(0.98,1.77) | 0.069 |
| 3000-3499 | 1.16(0.94,1.34) | 0.124 |  | 1.13(0.96,1.34) | 0.131 |  | 1.25(0.96,1.63) | 0.103 |
| 2500-2999 | Reference |  |  | Reference |  |  | Reference |  |
| <2500 | 0.84(0.63,1.13) | 0.258 |  | 1.10(0.78,1.54) | 0.593 |  | 1.51(0.97,2.35) | 0.071 |
| Notes: Adjusted for sex, residence area, gestational age, fetus number, feeding pattern, single-child or not, paternal overweight/obesity, maternal overweight/obesity, paternal educational level, maternal educational level, daily fruit and vegetable consumption, daily meat consumption, daily SSBs consumption and daily moderate to vigorous physical activity. | | | | | | | | |

| Table S3 Risk of childhood abdominal obesity in boys and girls with different birth weights in participants with original birth weight records(N=21,615) | | | | | |
| --- | --- | --- | --- | --- | --- |
| Groups | Crude model | |  | Adjusted model | |
|  | OR (95%CI) | *P* value |  | OR (95%CI) | *P* value |
| Total |  |  |  |  |  |
| ≥4000 | 1.49(1.34,1.65) | < 0.001 |  | 1.37(1.23,1.53) | < 0.001 |
| 3500-3999 | 1.31(1.21,1.43) | < 0.001 |  | 1.26(1.15,1.37) | < 0.001 |
| 3000-3499 | 1.10(1.02,1.20) | 0.013 |  | 1.08(1.00,1.18) | 0.065 |
| 2500-2999 | Reference |  |  | Reference |  |
| <2500 | 1.00(0.85,1.18) | 0.996 |  | 0.95(0.80,1.13) | 0.569 |
| Boys |  |  |  |  |  |
| ≥4000 | 1.58(1.37,1.82) | < 0.001 |  | 1.52(1.30,1.77) | < 0.001 |
| 3500-3999 | 1.35(1.19,1.52) | < 0.001 |  | 1.35(1.18,1.54) | < 0.001 |
| 3000-3499 | 1.21(1.07,1.36) | 0.002 |  | 1.22(1.07,1.39) | 0.002 |
| 2500-2999 | Reference |  |  | Reference |  |
| <2500 | 1.06(0.83,1.34) | 0.661 |  | 1.05(0.81,1.36) | 0.706 |
| Girls |  |  |  |  |  |
| ≥4000 | 1.41(1.21,1.65) | < 0.001 |  | 1.27(1.08,1.49) | 0.004 |
| 3500-3999 | 1.30(1.16,1.46) | < 0.001 |  | 1.20(1.06,1.36) | 0.003 |
| 3000-3499 | 1.03(0.92,1.15) | 0.600 |  | 0.99(0.88,1.11) | 0.814 |
| 2500-2999 | Reference |  |  | Reference |  |
| <2500 | 0.96(0.76,1.20) | 0.957 |  | 0.88(0.70,1.13) | 0.334 |
| Notes: crude model was adjusted for residence area, age and birth weight record method; the adjusted model was further adjusted for gestational age, fetus number, feeding pattern, single-child or not, paternal overweight/obesity, maternal overweight/obesity, paternal educational level, maternal educational level, daily fruit and vegetable consumption, daily meat consumption, daily SSBs consumption, daily moderate to vigorous physical activity and birth weight record method on the basic of crude model. | | | | | |

| Table S4 Associations between birth weight and childhood abdominal obesity in participants with original birth weight records, stratified by age groups | | | | | | | | |
| --- | --- | --- | --- | --- | --- | --- | --- | --- |
| Birth weight | 6-9 years old | | 10-13 years old | | |  | 14-17 years old | |
| groups | OR (95% CI) | *P* value |  | OR (95% CI) | *P* value |  | OR (95% CI) | *P* value |
| ≥4000 | 1.43(1.20,1.701) | < 0.001 |  | 1.34(1.11,1.60) | < 0.001 |  | 1.35(1.06,1.72) | 0.013 |
| 3500-3999 | 1.19(1.03,1.37) | 0.015 |  | 1.33(1.16,1.54) | < 0.001 |  | 1.22(1.00,1.50) | 0.053 |
| 3000-3499 | 1.01(0.88,1.15) | 0.885 |  | 1.13(0.98,1.29) | 0.086 |  | 1.13(0.93,1.38) | 0.217 |
| 2500-2999 | Reference |  |  | Reference |  |  | Reference |  |
| <2500 | 0.85(0.65,1.12) | 0.251 |  | 0.95(0.71,1.27) | 0.735 |  | 1.22(0.83,1.79) | 0.309 |
| Notes: Adjusted for sex, residence area, gestational age, fetus number, feeding pattern, single-child or not, paternal overweight/obesity, maternal overweight/obesity, paternal educational level, maternal educational level, daily fruit and vegetable consumption, daily meat consumption, daily SSBs consumption and daily moderate to vigorous physical activity and birth weight record method. | | | | | | | | |
